# Biomechanical analysis of costochondral graft fracture in temporomandibular joint replacement

**DOI:** 10.1038/s41598-020-74548-1

**Published:** 2020-10-20

**Authors:** Yi Mao, Xuzhuo Chen, Shiqi Yu, Weifeng Xu, Haiyi Qin, Jinze Zhen, Yating Qiu, Shanyong Zhang, Chi Yang

**Affiliations:** 1 Department of Oral Surgery, Ninth People’s Hospital, College of Stomatology, Shanghai Jiao Tong University School of Medicine, and Shanghai Key Laboratory of Stomatology & Shanghai Research Institute of Stomatology, Shanghai, 200011 China; 2 Shanghai Ninth People’s Hospital, School of Biomedical Engineering, Shanghai Jiao Tong University, Shanghai, 200011 China; 3 National Die and Mold CAD Engineering Research Center, Shanghai Jiao Tong University, Shanghai, 200011 China

**Keywords:** Dental diseases, Oral diseases

## Abstract

This study is the first attempt to explore the reason of costochondral graft fracture after lengthy mandible advancement and bilateral coronoidectomy by combining finite element analysis and mechanical test. Eleven groups of models were established to simulate costochondral graft reconstruction in different degrees of mandible advancement, ranging from 0 to 20 mm, in 2 mm increment. Force and stress distribution in the rib-cartilage area were analyzed by finite element analysis. Mechanical test was used to evaluate the resistance of the rib-cartilage complex. Results showed a sharp increase in horizontal force between 8 and 10 mm mandible advancement, from 26.7 to 196.7 N in the left side, and continue increased after 10 mm, which was beyond bone-cartilage junction resistance according to mechanical test. Therefore, we concluded that bilateral reconstruction with coronoidectomy for lengthy mandible advancement (≥ 10 mm) may lead to prominent increase in shear force and result in a costal-cartilage junction fracture, in this situation, alloplastic prosthesis could be a better choice. We also suggested that coronoidectomy should be carefully considered unless necessary.

## Introduction

Costochondral graft (CCG) has been a mainstream method for mandibular reconstruction in a long time, especially before artificial joints were developed^1–5^. The native size and morphological similarity make it a perfect match in TMJ. In addition, growth potential and minimal immunological counter-response also contribute to the advantages of CCG^6,7^. Complications including ankyloses, overgrowth, graft resorption, postoperative pain, donor site morbidity and osteolysis^1–3,5,8,9^. Several reports^10,11^ mentioned postoperative costochandral graft fracture, usually occurred in cases with coronoidectomy, but the fracture reason so far has not been explored.

Patients suffered from Idiopathic condylar resorption (ICR) tend to exhibit a decrease in posterior facial height, retrognathism, and progressive anterior open bite with a clockwise rotation of the mandible^12–14^. For active ICR, condylectomy and CCG reconstruction is a considerable choice. Troulis et al.^15,16^ reported long-term stability of CCG in ICR and all of twenty-six patients achieved stable results. Alloplastic prosthesis has the advantage of avoiding secondary surgery, but the price is very expensive, limiting the use in clinical especially in developed countries.

**Figure 1:**
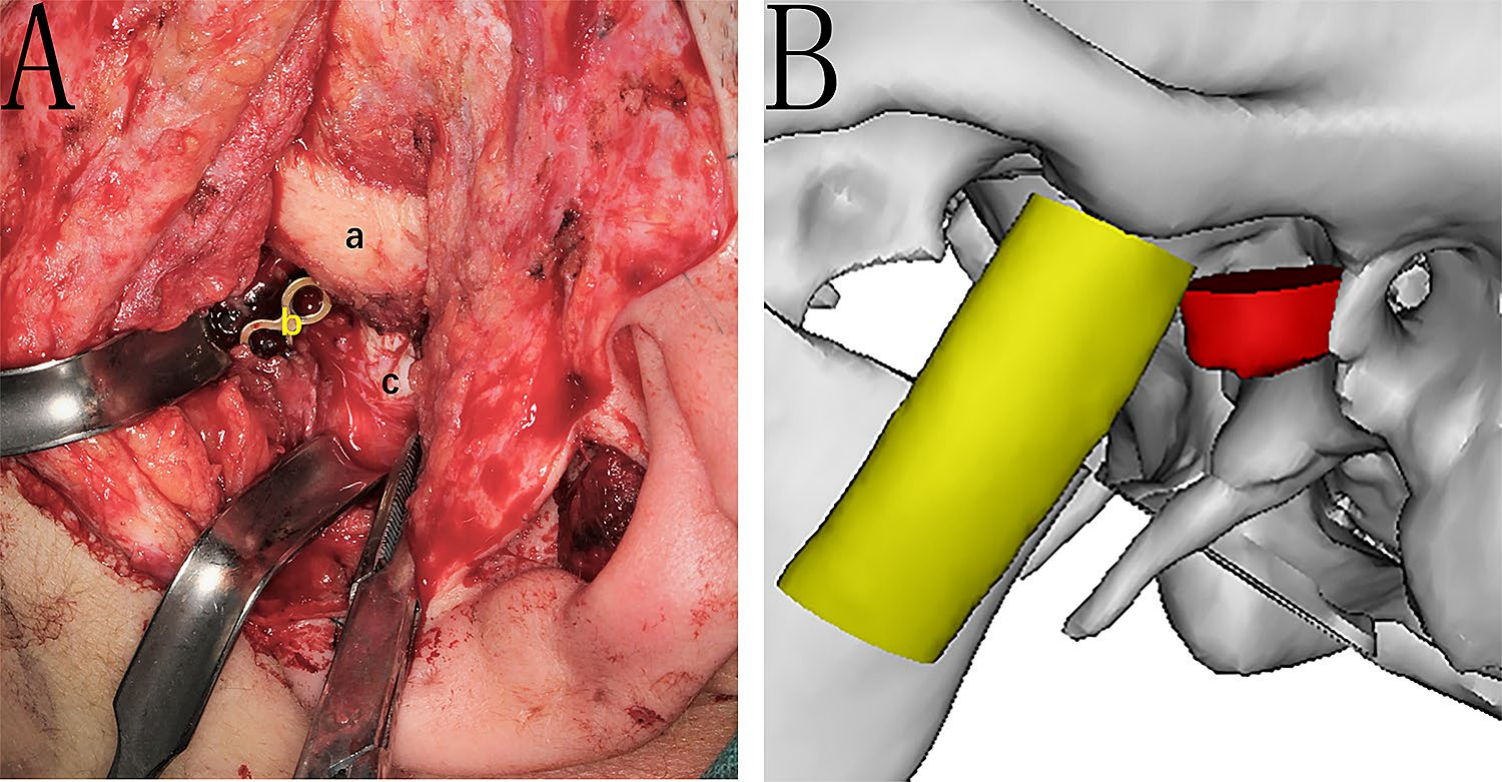
Photo during second time operation, costochondral graft was fractured from the junction. (**A**): the cartilage was broken from the rib and displaced to the back. a: Zygomatic arch, b: rib with titanium plate, c: rib cartilage. (**B**): simulated images: the cartilage was broken from the rib and displaced to the back.

We have performed a CCG reconstruction for a 25-year-old Chinese lady who diagnosed as ICR through detailed clinical and imaging examination. The mandible was advanced approximately 13 mm to achieved a normal occlusion, bilateral coronoidectomy was performed Simultaneously to avoid mouth opening limitation. Unfortunately, relapse happened within 6 months, the patient presented with facial asymmetry and occlusal disorders. At first we thought the ribs had been absorbed, but during the second surgery, we found the patient’s graft had fractured from the bone-cartilage junction (Fig. 1). Merkx and Freihofer^11^ inferred that a significant muscular component might contribute to this disappointing result, but they did not verify it. We hypothesized that lengthy mandible advancement with coronoidectomy may alter force distribution on the CCG, which beyond CCG’s resistance, hence lead to rib fracture. This study was to explore the reason of CCG fracture by finite element analysis (FEA) and mechanical test.

## Results

### Stress distribution

**Figure 2:**
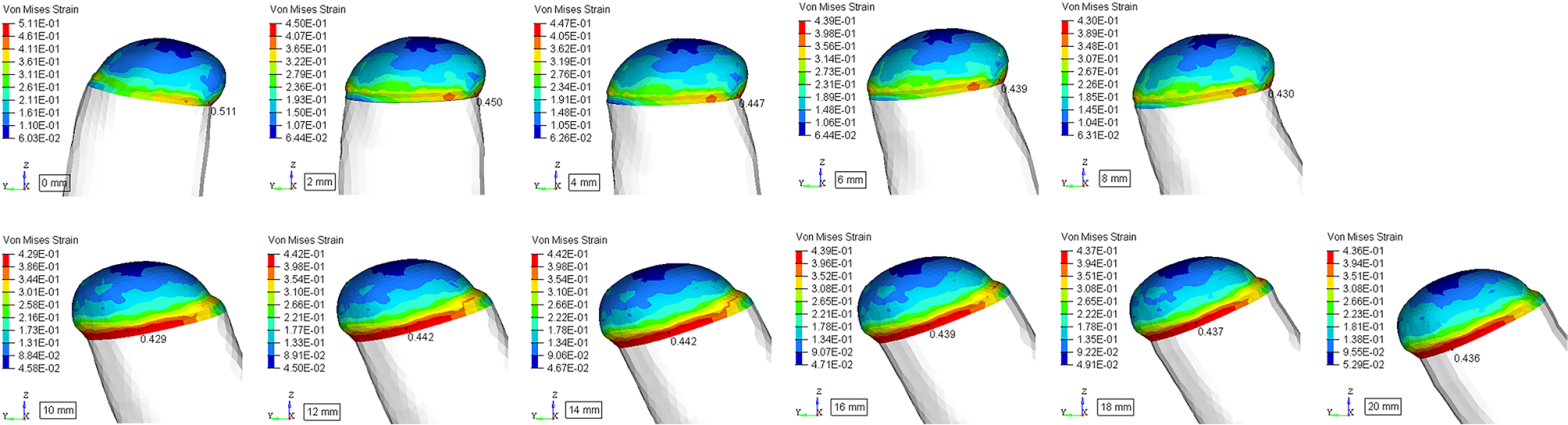
Stress and strain distribution on the CCG. From 0 to 8 mm, cartilage deformed forward, stress slightly concentrated on the anterior edge of the neck; from 10 to 20 mm, cartilage deformed backward, stress heavily concentrated on the posterior edge of the neck.

Obvious distortion of the rib cartilage was observed after muscle force loading. when mandible advancement ranged from 0 to 8 mm, costal cartilage deformed forward, stress was slightly concentrated on the anterior edge of the neck. However, when the mandible advanced more than 10 mm, rib cartilage deformed backward, stress was heavily concentrated on the posterior edge of the neck (Fig. 2). Cartilage was likely to break on the posterior margin of the neck from 10 to 20 mm.

### Force alteration

**Figure 3:**
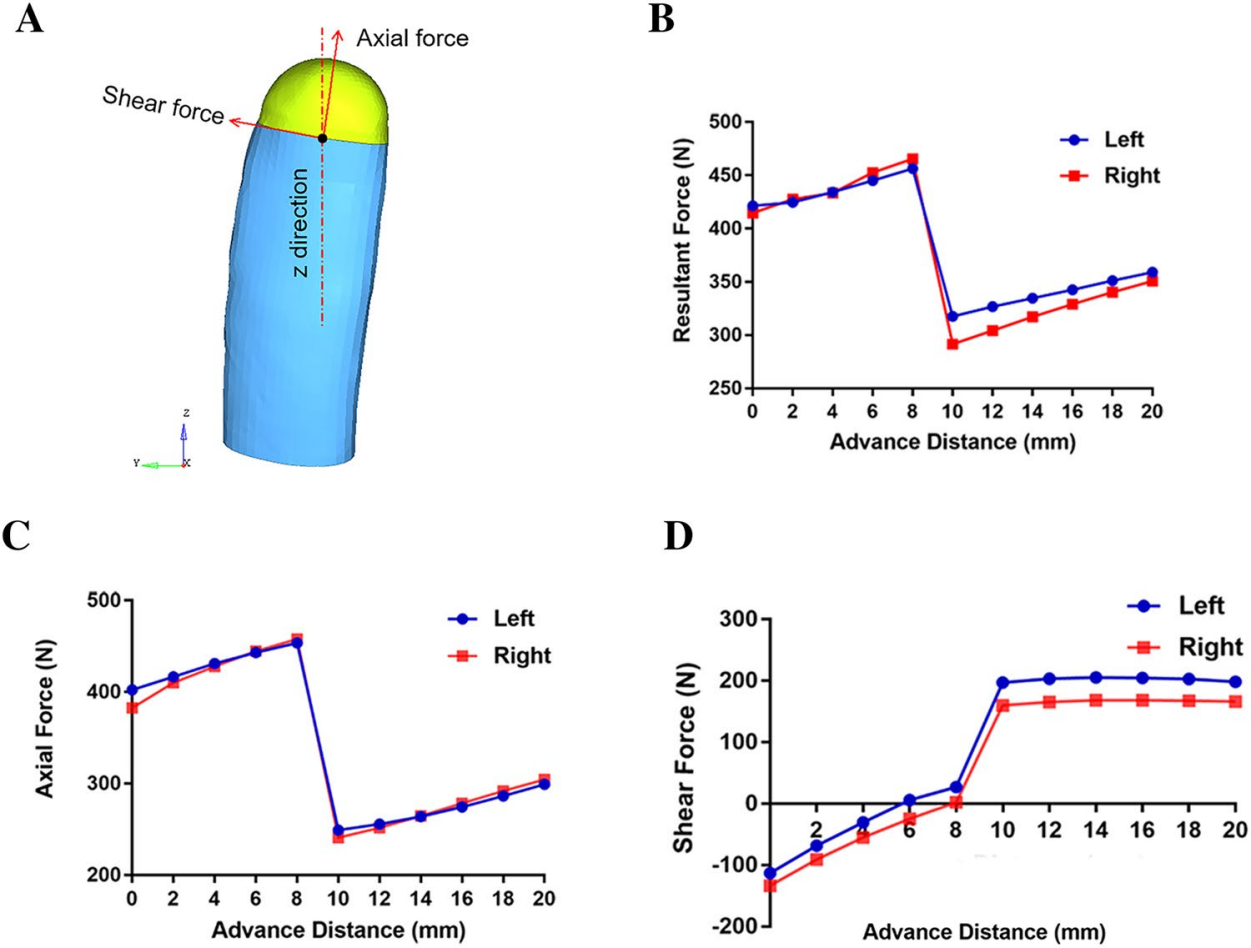
Force alteration trend of resultant force, axical force, and shear force. (**A**) Diagram of the decomposition of resultant forces into axial and shear forces. (**B**) Alteration trend of resultant force. (**C**) Alteration trend of axial force. (**D**) Alteration trend of shear force.

To better analysis force alteration during mandibular advancement, we decomposed the resultant force into axial force parallel on the long axis, and shear force perpendicular on the long axis (Fig. 3). Corresponding data were shown in Table 1. Prominent force changes took place between 8 and 10 mm advancements.

**Table 1:** Data of the resultant force, shear force and axial force at each point.

	Resultant force (N)			Vertical force (N)			Shear force (N)		
	Left	Right	Average	Left	Right	Average	Left	Right	Average
0 mm	421.3	414.7	418.0	402.3	382.7	392.5	− 113.2	− 133.7	− 123.5
2 mm	424.7	427.7	426.2	416.7	410.1	413.4	− 68.7	− 91.1	− 79.9
4 mm	434.2	433.4	433.8	431.0	427.9	429.5	− 30.7.	− 55.3	− 43.0
6 mm	445.0	452.5	448.8	443.2	444.5	443.9	5.6	− 24.9	− 9.7
8 mm	456.3	465.6	461.0	453.6	457.9	455.8	26.7	1.9	14.3
10 mm	317.6	291.5	304.6	249.2	240.8	245.0	196.7	159.6	178.2
12 mm	326.7	304.3	315.5	255.6	251.7	253.7	203.1	165.4	184.3
14 mm	334.6	317.2	325.9	263.9	264.6	264.3	205.3	168.3	186.8
16 mm	342.6	329.0	335.8	274.4	278.4	276.4	204.5	168.2	186.4
18 mm	351.2	340.3	345.8	286.4	291.8	289.1	202.6	167.1	184.9
20 mm	359.0	350.8	354.9	299.0	304.4	301.7	198.0	165.9	182.0

#### Resultant force behavior

When the mandible advanced from 0 to 8 mm, the resultant force on the connector increased in a linear fashion, from 421.3 N to 456.3 N in the left, from 414.7 N to 465.6 N in the right, respectively. There was a significant reduction (138.7 N in the left, 174.1 N in the right) between 8 and 10 mm, where the coronoid process had just been excised, from 456.3 N to 317.6 N in the left, from 465.6 N to 291.5 N in the right, respectively. With the mandible continue advancing from10mm to 20 mm, the force increased again in a linear fashion, from 317.6 N to 359.0 N in the left, from 291.5 N to 350.8 N in the right, respectively (Fig. 3, Table 1).

#### Axial force behavior

When the mandible advanced from 0 to 8 mm, the axial force on the connector increased in a linear fashion, from 402.3 N to 453.6 N in the left, 382.7 N to 457.9 N in the right, respectively. There was a significant reduction (204.4 N in the left, 217.1 N in the right) between 8 mm and the 10 mm, where the coronoid process had just been excised, from 453.6 N to 249.2 N in the left, 457.9 N to 240.8 N in the right, respectively. With the mandible continue advancing from10mm to 20 mm, the force increased again in a linear fashion, from 249.2 N to 299.0 N in the left, 240.8 N to 304.4 N in the right, respectively (Fig. 3, Table 1).

#### Shear force behavior

When the mandible advanced from 0 to 8 mm, the shear force on the connector decreased in a linear fashion, from -113.2 N to 26.7 N in the left, -133.7 N to -1.9 N in the right, respectively. There was a significant increase (170 N in the left, 157.7 N in the right) between 8 mm and the 10 mm, where the coronoid process had just been excised, from 26.7 N to 196.7 N in the left, 1.9 N to 159.6 N in the right, respectively. With the mandible continue advancing from10 to 20 mm groups, there was no significant change in shear force, stayed around 200 N in the left, 168 N in the right, respectively (Fig. 3, Table 1).

### Mechanical tests

The mean maximum shear and axial forces a costal cartilage could withstand were approximately 192 N and 1898 N, respectively (Table 2).

**Table 2:** Maximum resistance of CCG samples, Horizontally (n = 5) and vertically (n = 5).

Number	Horizontal resistance (N)	Number	Vertical resistance (N)
NO. 1	169	NO. 6	2058
NO. 2	197	NO. 7	1969
NO. 3	199	NO. 8	1446
NO. 4	207	NO. 9	2020
NO. 5	188	NO. 10	1998
Average	192	Average	1898

### Shear Force alteration with or without coronoidectomy

The data of shear force alteration from 0 to 8 mm with or without coronoidectomy was showed in Table 3. Without coronoidectomy, the shear force decreased in a linear fashion, from -113.2 N to 26.7 N in the left, from -133.7 N to -1.9 N in the right, respectively. With coronoidectomy, it increased in a linear fashion, from 94.6 N to 188.5 N in the left, from 70.1 N to 157.7 N in the right, respectively.

**Table 3:** Shear force value with or without coronoid process at each point.

Shear force	Left		Right	
	With	Without	With	Without
0 mm	− 113.2	94.6	− 133.7	70.1
2 mm	− 68.7	128.3	− 91.1	109.7
4 mm	− 30.7	150.7	− 55.3	126.9
6 mm	5.6	170.7	− 24.9	144.0
8 mm	26.7	188.5	1.9	157.7

## Discussion

In 1920, Gillies firstly described the use of costochondral graft(CCG) to perform a Temporomandibular joint(TMJ) reconstruction, but it didn't become popular until 1974^17^. Since then, CCG has been a mainstream method in a long time, especially before artificial joints were developed^1–5^. CCG has a cartilaginous cap, the native size and morphological similarity make this type of graft a perfect match in TMJ. In addition, growth potential and minimal immunological counter-response also contribute to the advantages of CCG^6,7^. Scholars including Awal et al.^1^ (N = 74), Kumar et al.^2^ (N = 6), Medra^4^ (N = 85), Perrott et al.^5^ (N = 33), reported the outcomes of long-time effects, of all their studies, ankyloses^3^, overgrowth, and resorption were major components of complications. Other complications including postoperative pain, donor site morbidity^8^ and osteolysis^9^. It is worth noting that several reports^10,11^ mentioned postoperative costochandral graft fracture, mostly occurred at CCG-ramus junction.

Idiopathic condylar resorption (ICR) can occur with a variety of underlying factors^14^. It is mostly bilateral and seems to have a high occurrence in women aged 15 to 35, especially those with a preexisting temporomandibular joint (TMJ) dysfunction and a high mandibular plane angle. As the result, these patients tend to exhibit a decrease in posterior facial height, retrognathism, and progressive anterior open bite with a clockwise rotation of the mandible^12–14^. Several therapies had been proposed to treat ICR at different stages^18–23^. For patients with active ICR, condylectomy and CCG reconstruction is a considerable choice. Troulis et al.^15,16^ reported long-term stability of CCG in ICR, twenty-six patients were involved and achieved a stable result.

Alloplastic prosthesis has the advantage of avoiding secondary surgery, but the price is very expensive. Besides, only one type of prosthesis has license in China by now. Based on the above facts, we performed a CCG reconstruction for this present patient who diagnosed as ICR through Detailed clinical and imaging examination. The mandible was advanced approximately 13 mm to achieved a normal occlusion, bilateral coronoidectomy was performed Simultaneously to avoid mouth opening limitation. Unfortunately, relapse happened within 6 months, the patient presented with facial asymmetry and occlusal disorders. During the second surgery, we found the patient’s graft had ruptured from the bone-cartilage junction. Merkx and Freihofer^11^ inferred that a significant muscular component might contribute to this disappointing result, but they did not verify it. We hypothesized that lengthy mandible advancement with coronoidectomy may alter force distribution on the CCG, hence lead to rib fracture. To explore the truth, two questions must be answered: one is the force distrubution of CCG, the other is the resistance of CCG, therefore, we conducted an FEA and mechanical test as the above. As far as we know, this is the first trial to explain the reason of CCG fracture, and also the first study to combine FEA with mechanical testing of the temporomandibular joint.

As lateral pterygoid has been cut during surgery, the shear force on the CCG neck consisted of three parts: horizontal force of temporalis, medial pterygoid and masseter. Only the temporalis muscle had a backward component in the horizontal direction, the other two muscles directed forward. The balance of these three muscles determined the direction and value of shear force.

According to our experiment, when mandible advancing in the range from 0 to 8 mm, shear force directed backward, so the new condyle was acted upon by a forward and downward force from the posterior wall of the glenoid fossa, and deform forward (Fig. 2). During mandible advancing, the shear force was gradually decreased and always within safe range (Table 1). However, with the continual advancement of mandible, the coronoid progress could contact with the inner face of zygomatic bone and limit mouth opening. Therefore, coronoidectomy was performed from 10 mm advancement. Without temporalis, the former muscle force balance has been interrupted and only medial pterygoid and masseter was left. Then all the related muscles directed forward in the horizontal direction, CCG deformed backward by contacting with the anterior wall of the articular fossa, and the value of shear force increased steeply. It could be over 196.7 N (Table 1) once advancing more than 10 mm and beyond CCG’s resistance, which was about 192 N according to the mechanical test (Table 2). Hence we concluded that bilateral CCG reconstruction with coronoidectomy for large extent mandible advancement lead to a sharp increase in horizontal component, resulting in CCG fracture at the bone-cartilage junction.

During mandible advancement process, horizontal component was affected by both magnitude and angle of muscle force. To find out whether the dramatic change in horizontal component between 8 and 10 mm was due to the loss of temporal muscle or the change in angle, we compared the distribution of shear force values after and without coronoidectomy from 0 to 8 mm (Table 3). The results showed that after coronoidectomy, there would be an increased forward shear force on the CCG neck from 94.6 N to 188.5 N in the left, 70.1 N to 157.7 N in the right, respectively. By contrast, without coronoidectomy the shear force directed backward and decreased from 113.2 N to 26.7 N in the left, 133.7 N to 1.9 N in the right, respectively. It confirmed that loss of temporalis is the main cause of prominent shear force increase between 8 to 10 mm. Besides, bilateral CCG reconstruction with coronoidectomy for mandible advancement in a small range (< 8 mm) still remained safe, which supports the fact that CCG is a mature method in the last few decades.

Autogenous bone grafts and alloplastic prosthesis were widely used for TMJ reconstruction, each with its own advantages. Controversies about these two methods exited for a long time^24–26^. So far, there has been no clear conclusion as to which conditions are more suitable for artificial joints. This present study suggested that the strength of CCG is not strong enough to counteract muscle strength during extensive mandibular advancement and coronal process resection, resulting in a period of postoperative instability. Some researchers have reported that the mechanical properties of artificial joints^27,28^. According to the prosthesis manual issued by TMJ Concepts Company, the average yield strength was 3514 N, which is far more resistant than CCG, enough to withstand horizontal shear forces. From this point of view, for patients with severe mandibular retraction, the artificial joint is more suitable for TMJ reconstruction.

Our study has some limitations that must be addressed: the CT data was from one specific patient while the maximal muscle forces were from literature, so the loading value can hardly accurately reflect the reality of the situation. Furthermore, the rib in mechanical test was not from human being, even though the size and cartilage height were almost the same as the pork rib, the actual rib resistance might be slightly different. Despite those shortcomings, mechanical change trends can be well simulated.

In conclusion, bilateral CCG reconstruction with coronoidectomy for mandible advancement in a large degree (≥ 10 mm) will induce a prominent force alteration and likely lead to CCG fracture, for this situation, alloplastic prosthesis should be a better choice. In addition, the coronal process plays an important role in maintaining joint balance and should not be easily detached.

## Methods

All methods were carried out in accordance with relevant guidelines and regulations. All experimental protocols were approved by Shanghai Ninth People’s Hospital Ethical Committee (SH9H-2019-T288-1). Informed consent has been obtained in this study.

### Establishment of computer-aided design (CAD) models

CT data were obtained from the relapsed patient, a 25-year-old female who suffered from condylar resorption several times without clear etiology (slice thickness, 0.625 mm; GE Healthcare, Buckinghamshire, England). Data were stored and then imported into Mimics software (Version 20.0, Medical, Leuven, Belgium) for 3D reconstruction. The surgical procedure was simulated in a 3D model. Contralateral seventh rib was selected and cut into suitable size, the cartilage side was contacted with the middle of glenoid fossa, the bone side was smoothly contact with the posterior margin of mandible ramus.

**Figure 4:**
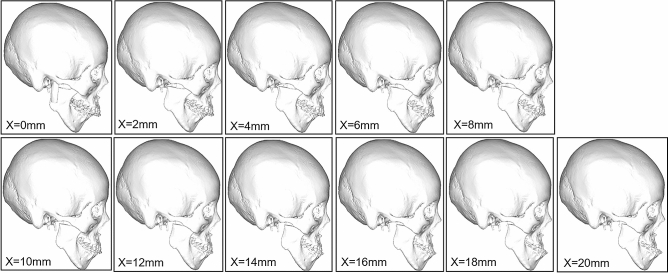
Eleven groups of condylectomy and costochondral graft reconstruction models were established between 0 and 20 mm advancement in 2 mm increments, that is, 0 mm, 2 mm, 4 mm, 6 mm, 8 mm, 10 mm, 12 mm, 14 mm, 16 mm, 18 mm, and 20 mm groups, respectively.

**Figure 5:**
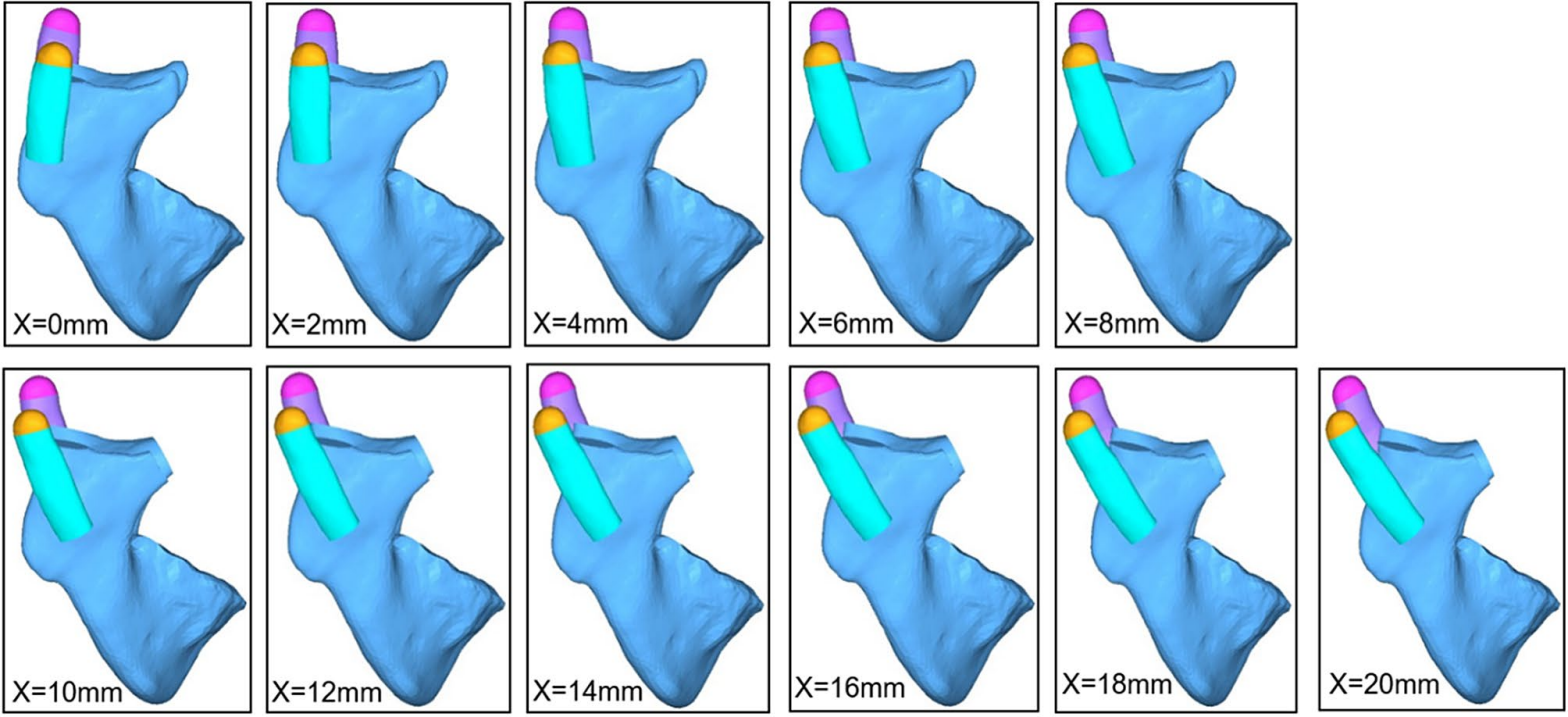
To avoid the inner face of frontal part of zygomatic bone blocks the coronoid process, coronectomy was performed from 10 to 20 mm groups.

In order to simulate the different distances of mandibular advancement, eleven groups of models were established advancing from 0 to 20 mm in 2 mm increments (Fig. 4). It is worth noting that once mandible advanced more than 8 mm, mouth opening would be limited as the inner face of the frontal section of the zygomatic bone blocks the coronoid process, so coronoidectomy must be performed from 10 mm and over groups (Fig. 5).

### Establishment of finite element model

Finite element models (FEM) are key tools which can be applied to mandible biomechanics^29^. We used Hypermesh, a finite element grid generation module software by Hyper Works, for pre-processing. LS-DYNA, the dynamic explicit algorithm module software from LSTC software, was used as the solver and processor.

**Figure 6:**
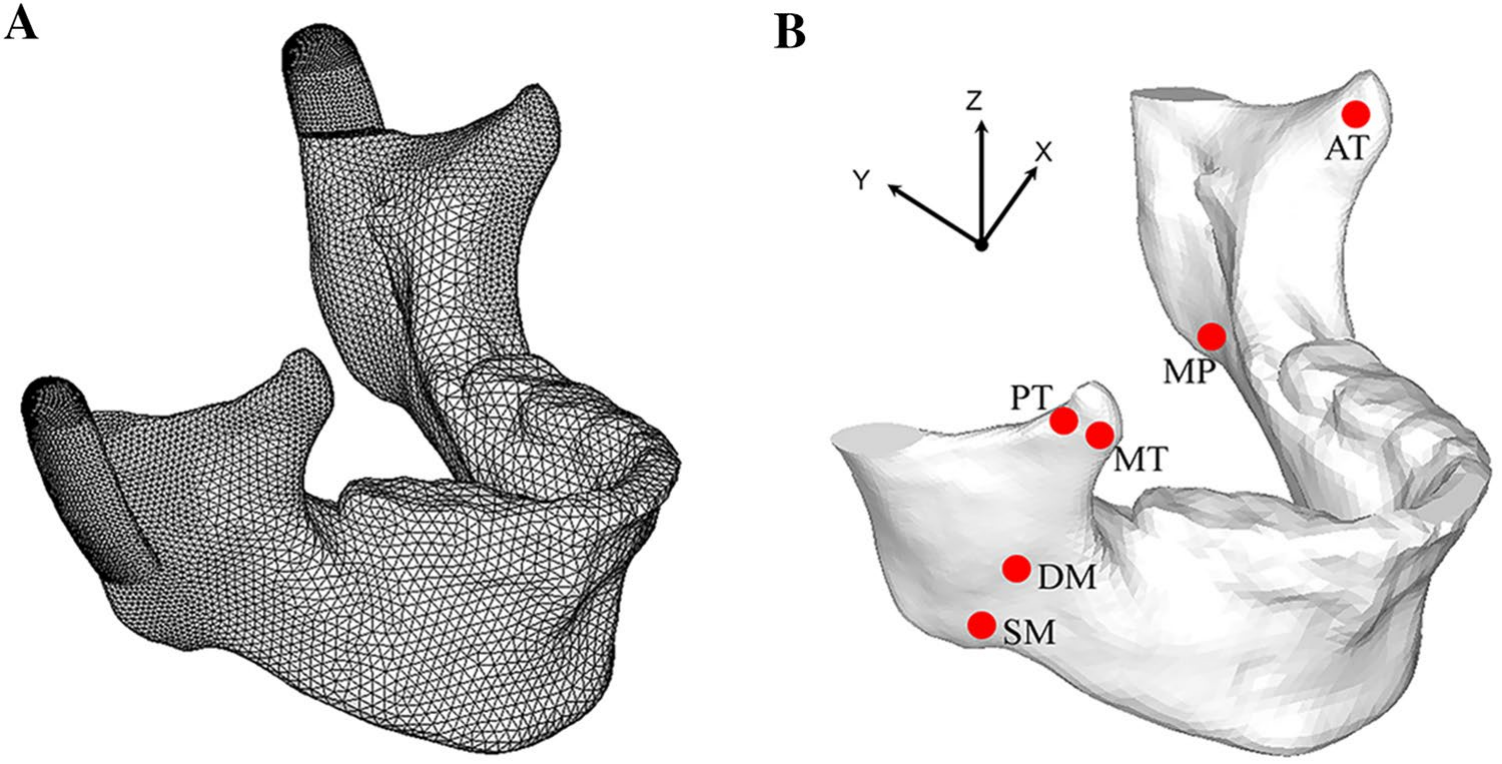
(**A**) Tetrahedral mesh was used in the analysis while the locations of the prosthesis and areas with large deformation were treated with mesh densification. (**B**) muscle attachment of temporalis (AT, PT, MT), masseter (DM, SM), and medial pterygoid (MP).

Analysis only included skull regions in contact with the CCG. Distant regions were simplified by applying boundary conditions. Tetrahedral mesh was used in the analysis while locations of the prosthesis and areas with large deformation were treated with mesh densification. The average mesh size of the mandibular was 2 mm. For locations with large curvature and possibly large stress, the mesh size of 1 mm was used for intensify. The average mesh size for rib was 0.8 mm, and the costochondral cartilage in contact with the articular fossa was 0.4 mm. The whole calculation model has a total of 45,700 elements and 155,200 nodes (Fig. 6a).

Computation can be reduced by simplifying screw connections. In our analysis, the rigid deformation mode of the tetrahedral element and low calculation accuracy were resolved by using the selective reduction integral form with node rotation for the tetrahedral element.

### Material parameters and boundary conditions

In the FEA, the ‘engineering stress–strain curve’ of the material was converted into the ‘real stress–strain curve’. Converted material data^30^ were listed in Table 4.

**Table 4:** Material parameters of costochondral graft.

	Cartilage	Bone
Density 1.0e^3^ kg/m^3^	2.0	1.8
Elasticity modulus/GPa	0.08	15
Poisson ratio	0.45	0.35
Engineering yield strength (*σ*_*s*_)/MPa	1.5	135
Engineering tensile strength (*σ*_*b*_)/MPa	2.4	150
Ductility %	40	1
True tensile strength (*σ*_*b*_)/MPa	3.4	152
True fracture strain %	34	1

To ensure the stability of the bone graft, the stress of each part of the complex should be less than the yield strength of the material. Following related references, we exerted maximum muscle force on the models to simulate extreme conditions. Parameters^29,31^ of six principal jaw-closing muscles were listed in Table 5. The six main muscles involved: superficial masseter(SM), deep masseter(DM), medial pterygoid(MP), anterior temporalis(AT), middle temporalis(MT), posterior temporalis(PT) were simulated to both sides of the mandible from corresponding muscle attachment (Fig. 6b).

**Table 5:** Maximum force value of involved muscles.

	Left (N)				Right (N)			
	F	Fx	Fy	Fz	F	Fx	Fy	Fz
SM	190.4	− 39.4	− 79.8	168.3	190.4	39.4	− 79.8	168.3
DM	81.6	− 44.6	29.2	61.9	81.6	44.6	29.2	61.9
MP	132.8	64.6	− 49.6	105.1	132.8	− 64.6	− 49.6	105.1
AT	154.8	− 23.1	− 6.8	153	154.8	23.1	− 6.8	153
MT	91.8	− 20.4	45.9	76.8	91.8	20.4	45.9	76.8
PT	71.1	− 14.8	60.8	33.7	71.1	14.8	60.8	33.7

SM = Superficial masseter; DM = Deep masseter; MP = Medial pterygoid; AT = Anterior temporalis; MT = Middle temporalis; PT = Posterior temporalis.

As the mandible moves forward, the muscle length and strength changed, based on the formula: F = PAi (F = muscle force; P = the intrinsic muscle strength constant, P = 0.37*106 N; A = the physiological cross-sectional area of the muscle) with the constant muscle volume, we deduced the formula: Fx = F_0_*L_0_/L_X_, enabling the calculation of direction and value of muscle forces (Table 6).

**Table 6:** Muscular forces value at each degree of mandible advancement.

	X(mm)	0	2	4	6	8	10	12	14	16	18	20
Left (N)	SM	190.40	194.40	198.15	202.04	205.18	208.41	211.27	214.20	216.20	218.24	219.28
	DM	81.60	84.59	87.82	90.70	93.45	96.38	98.79	100.59	102.45	103.60	103.99
	MP	132.80	135.86	139.07	142.06	144.77	147.19	148.84	150.53	151.83	152.26	152.70
	AT	154.80	153.35	151.58	149.85	147.83						
	MT	91.80	90.18	88.45	86.61	84.70						
	PT	71.10	69.30	67.46	65.71	63.94						
Right (N)	SM	190.40	195.74	199.34	203.07	206.50	209.60	212.34	215.14	217.54	219.49	220.98
	DM	81.60	84.31	86.74	89.32	91.55	93.89	96.35	98.36	100.15	101.69	102.63
	MP	132.80	136.42	140.24	143.87	147.25	150.35	153.11	155.48	156.95	158.44	158.94
	AT	154.80	153.37	151.62	149.57	147.25						
	MT	91.80	90.17	88.60	86.94	85.19						
	PT	71.10	69.55	67.82	66.29	64.71						

Muscle values at each point, calculated based on Fx = F_0_*L_0_/L_x_. Once mandible advanced more than 10 mm, AT, MT, PT disappeared with coronoidectomy.

Bonded contact relationship was established between the CCG and the mandible. The load was set to anterior teeth, the fixed constraint set to zygomatic arch and lower incisor to mimic the biting movement.

### Mechanical tests

In order to verify whether stress changes in joint areas were sufficient to cause the rib fracture, a mechanical test was performed, using a screen display electro-hydraulic universal testing machine (Guangcai test instrument limited company from Guangzhou, China).

**Figure 7:**
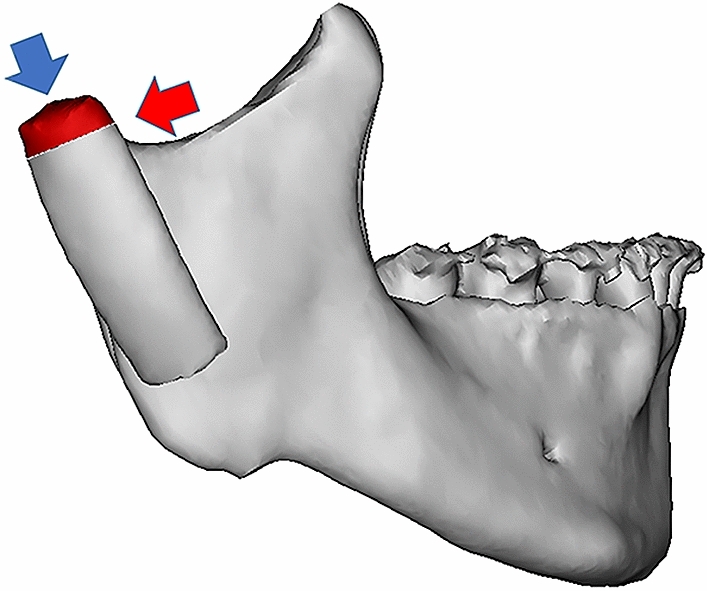
Apply forces to the ribs from two directions: Parallel to the long axis to test axial force resistance (blue arrow); Parallel to the costal-cartilage junction to test shear force resistance (red arrow).

We bought ten ribs from the market, about 10 mm wide at the neck. Cartilages were trimmed to 5 mm in height and evenly divided into two groups. In one group, the ribs were broken by loading shear forces parallel to the costal cartilage junction. In another group, the ribs were broken by loading axial force perpendicular to the costal cartilage (Fig. 7). The loading force was gradually increased until the cartilage was damaged or broken, at which point the force value was recorded.

### Comparing the force alteration with or without coronoidectomy

Add five groups of models: bilateral CCG reconstruction with coronoidectomy for mandible advancement in 0 mm, 2 mm, 4 mm, 6 mm, 8 mm, respectively. Only masseter and medial pterygoid muscle force were simulated, FEA was then performed. The results were compared with bilateral CCG reconstruction without coronoidectomy at the same mandibular advancing degree.
